# Exposure to Quartz in Finnish Workplaces Declined during the First Six Years after the Signing of the NEPSI Agreement, but Evened out between 2013 and 2017

**DOI:** 10.3390/ijerph15050906

**Published:** 2018-05-03

**Authors:** Tapani Tuomi, Markku Linnainmaa, Sirpa Pennanen

**Affiliations:** Finnish Institute of Occupational Health, P.O. Box 40, FI-00032 TYÖTerveyslaitos, Finland; markku.linnainmaa@ttl.fi (M.L.); sirpa.pennanen@ttl.fi (S.P.)

**Keywords:** respirable crystalline silica, quartz exposure, NEPSI agreement, silicosis

## Abstract

To reduce the incidence of occupational diseases related to exposure to respirable silica at work, the main industries in the EU associated with respirable silica agreed on measures to improve working conditions through the application of good practices. These practices were included in “The Agreement on Workers Health Protection through the Good Handling and Use of Crystalline Silica and Products Containing it” (NEPSI agreement), signed in April 2006. In Finland, we have previously seen a decline in exposure to respirable quartz in relevant industries upon the treaty coming into effect, during the years 2006–2013. The present paper examines trends in exposure to respirable crystalline silica in Finland from 2006 to the end of 2017. In addition, we looked at changes in the number of exposed workers and the prevalence of silicosis and lung cancer associated with the exposure during the same period. The aim was to find out whether the decline in exposure previously recorded had continued, and whether this, in addition to the previously reported descent in exposure, was reflected in the amount and prevalence of occupational diseases associated with inhaling respirable quartz. In the present study, during the period 2013 to 2017 no further improvements were observed. The exposure remained at an average level of 20–50% of the current OEL_8h_. This is not necessarily sufficient to eliminate silicosis, lung cancer or other health effects associated with exposure to respirable silica in affected workplaces. To bring about further improvements in exposure, we suggest the present OEL_8h_ in Finland (0.05 mg/m^3^) and particularly in the many EU countries with an OEL_8h_ of 0.1 mg/m^3^ be lowered to 0.020–0.025 mg/m^3^. Secondly, branches outside of the NEPSI treaty where the number of exposed workers is increasing in Finland and possibly in some other EU countries as well, namely building and refinery industries, would be advised to sign the treaty. In addition, as a result of signing, good practices should be developed for work tasks where exposure to respirable silica is of concern in these industries.

## 1. Introduction

Crystalline silica, i.e., quartz, cristobalite and tridymite are abundant in rocks and minerals. Cristoballite and tridymite are particularly rare polymorphs in Finland, while approximately 12% of the Earth’s crust consists of quartz [1]. Substantial quantities of quartz can also be found in sand, sandstone, and granite, clay, shale, and slate [1]. Thus, products containing these minerals as main ingredients are usually rich in crystalline silica. Hence, occupational exposure to quartz is frequently an issue in many workplaces. In Finland, according to recent estimates, 50,000 workers from a total workforce of 2.7 million (1.8%) are exposed to quartz in significant quantities, some of which contract silicosis each year (the Register of Occupational Diseases in Finland, 2014).

Critical branches or tasks with respect to quartz exposure include mining activities, concrete industry, glass and porcelain industry, foundries, and building activities, such as stone masonry, facade renovation, sand blasting of buildings, tunneling, scrabbling, cutting, or drilling of stone and concrete [2,3]. In Finland, building activities are fairly intensive due to a continuous regional migration from rural areas to cities starting in the 1960s as well as from abroad to regional population clusters. Immigration to Finland has been increasingly frequent since joining the European Union (EU) in 1995. As building activities are presently on the rise, so are industries supporting building activities, including foundries, as well as the production of cement and concrete slabs, plates, tiles, and asphalt [4]. In addition, new mines and plants that enrich metals and minerals have opened in Finland over recent decades, and the production of metal ores and metal concentrates have increased approximately ten-fold from 2006 to 2016 [5]. With this in mind, it is not surprising that while quartz exposure has declined after the signing of the European treaty on “The Agreement on Workers Health Protection through the Good Handling and Use of Crystalline Silica and Products Containing it”, i.e., NEPSI agreement, the number of workers exposed to quartz has remained stable [6,7].

The health risks of crystalline silica come from breathing respirable dust (particle diameter < 10 µm, median diameter = 4.25 µm [8,9]. The dust penetrates the alveolar region of the lungs and may cause silicosis—usually a nodular pulmonary fibrosis—as well as lung cancer [3,10,11,12,13]. Respirable quartz particles cause oxidative damage in lung tissues due to oxygen radicals bound to their surface as well as by inducing the production of oxygen radicals by phagocytic and epithelial cells in response to quartz particles [14]. It has also been shown that quartz particles penetrating the lungs promote the manifestation of oxidative damages in lung tissue through the inhibition of the oxidoreductase glucose 6-phosphate. This inhibition is prevented by the antioxidant glutathione. However, quartz particles also seem to inhibit the pentose phosphate pathway critical for the regeneration of glutathione from its oxidized state glutathione disulfide [15]. Damages in lung tissue can be an effect of inflammation, but inflammation may also promote damages formed by other mechanisms [16]. In addition to oxidative damages, long-term exposure to respirable quartz may also lead to a build-up of quartz particles in macrophages. This deposition can be permanent upon long-term significant exposure, and the development of silicosis can therefore proceed even after the exposure has ended. Oxidative damages and the deposition of quartz particles in macrophages can lead to the formation of scar tissue in lungs, i.e., lung fibrosis or silicosis. Oxidative damages are also associated with DNA-damages and the formation of lung cancer. Lung cancer is more prominent in silicosis patients, the mechanisms of which has been studied extensively, but can also be attained independently [3,10,11,12,13]. Both diseases are, nevertheless, associated with high mortality rates [3].

In addition to silicosis and lung cancer, exposure to respirable crystalline silica has been linked to cancer of the esophagus, stomach, and intestines, as well as kidney cancer. However, more research is needed to establish mechanisms or rule out pathogenesis, particularly for other cancers than lung and kidney cancer [3]. The manifestation of health effects is dependent on the duration and significance of the exposure. Silicosis can develop in less than five years, when exposed to concentrations well above 0.05 mg/m^3^. Respectively, in concentrations below 0.05 mg/m^3^, the outbreak of silicosis can take up to ten years or considerably longer [17]. In addition to the concentration of respirable quartz, the prevalence of health implications upon exposure is dependent on the size distribution of the dust, the time passed from the formation of the particles, as well as smoking status and impurities present in the particles. Freshly formed particles usually carry more oxygen radicals than old dust and hence is considered more dangerous. Impurities bound to quartz particles can either increase their harmfulness (for instance clay and other compounds rich in aluminum) or protect from harmful outcomes, such as iron [18,19].

The signing of the NEPSI agreement coincided with a harmonization of the occupational exposure limits (OEL_8h_) in some European countries. In most EU countries (13 out of 25), the OEL_8h_ for quartz is currently 0.1 mg/m^3^. The OEL_8h_ for cristoballite and tridymite is 0.05 mg/m^3^ in 10 and 11 EU countries, respectively. In Finland, the OEL_8h_ for quartz, cristoballite and tridymite was adjusted from 0.2 mg/m^3^ to 0.05 mg/m^3^ in 2007. In USA, the statutory permissible exposure limit (PEL) of the Occupational Safety and Health administration (OSHA) for quartz, cristoballite and tridymite is currently 0.05 mg/m^3^. OSHA has, however, stated in a recent expert opinion that “OSHA also finds significant risk remaining at the current PEL, but considers a PEL of 0.05 mg/m^3^ to be the lowest level that can reasonably be achieved through use of engineering controls and work practices in most affected operations” [20]. Similar to OSHA, the EU Scientific Committee on Occupational Exposure Limits (SCOEL) has recommended an occupational exposure limit (OEL_8h_) below 0.05 mg/m^3^ and concludes that “an average respirable silica concentration of 0.02 mg/m^3^ reduces the prevalence of silicosis to about 0.25% or less [13]. In line with this, the American Society of Industrial Hygienists (ACGIH) recommend a Threshold Limit Value (TLV) of 0.025 mg/m^3^ for all forms of respirable crystalline silica.

Consequently, the current OEL_8h_ in Finland and the prevailing workplace concentrations based on a previous overview covering the years 1994–2013 [7] while significantly lower than before the NEPSI agreement, are not sufficiently low to ensure a tolerable prevalence of silicosis and/or cancer in exposed workers. This would require a level of less than one diseased person in 10,000 workers per year [21], or even less than 1 in 1,000,000 [22]. The aim of the present study was to find out whether the positive development in the time-weighed quartz exposure in Finnish workplaces reported between 2006 and 2013 has continued. In addition, we wanted to find out, whether the general descent in exposure is reflected in the prevalence of silicosis and lung cancer in exposed workers.

## 2. Methods

### 2.1. Sampling Sites

Altogether 451 air samples were collected mostly indoors, from the breathing zone of workers, with the aim of estimating average 8 h workday exposure of workers to respirable crystalline silica. Samples were taken during the years 2013–2017 at customer service assignments—occupational hygiene surveys or follow-up measurements—carried out by occupational hygienists from six regional offices of the Finnish Institute of Occupational Health (FIOH) across Finland. Samples originated from industrial workplaces.

### 2.2. Sampling

Sampling of respirable dust was performed according to CEN and ISO standards, as described previously [7,8,9]. Briefly, either cyclones (SKC aluminum, or SKC GS-3 nylon) or SKC IOM samplers with MultiDust foam inserts to were used to preselect the respirable fraction (median diameter approximately 4 µm). Samples were collected from the breathing zone of workers. Sampling was continued for a minimum of 4 h to estimate the average 8 h exposure of workers.

### 2.3. FTIR and XRD Methodology

Samples containing > 20% of calcite (*w*/*w*) as evaluated by the hygienist, were pretreated with hydrochloric acid and 2-propanol (Analysis grade, VWR Chemicals, Darmstadt, Germany) as described earlier to remove calcite [7].

For FTIR analysis, filters were ashed, the ash combined with KBr and pressed into pellets as previously described [7]. Blank samples as well as calibration and control standards were prepared in an identical way.

For XRD analysis, filters were ashed as previously described [7]. The samples were transferred to capped test tubes using 2-propanol. (3 × 5 mL). A splinter of wood was used to removed ash stuck to the crucibles. The tubes were wortexed carefully and treated in an ultra-sonic bath for a minimum of 10 min prior to filtering the suspension through a 25 mm 0.45 µm silver membrane (AG4502550, Millipore Corp, Burlington, MA, USA) wetted with 2 mL of 2-propanol. The suspension was filtered using suction after firstly pouring it into the filtering funnel and letting it stand for 1 min. The test tubes were rinsed with 2 mL of 2-propanol, and the 2-propanol suspension filtered as above. The silver membrane was transferred with the sample side up to watch glass containing 2 drops of 2% parlodion solution in amyl alcohol (SPI Chemicals, West Chester, PA, USA). After the parlodion solution had been transferred to the membrane, the membrane was left to evaporate on a teflon platform. After ca. 10 min. the dry silver membrane was transferred to a sample holder for XRD analysis. Blank samples, as well as calibration and control standards were prepared in an identical way.

### 2.4. Analysis by FTIR

Samples and standards were measured identically as described in NIOSH method 7602, as described previously [7,23]. Briefly, the IR spectra was measured in absorbance mode. The pellet was scanned from 1000 cm^−1^ to 600 cm^−1^ and the peaks 775 and 800 cm^−1^ were used to identify quartz. Quantification was based on the absorbance (peak height) at 800 cm^−1^, using the mean of four consecutive measurements. The quantitative limit of detection was 6.5 µg/sample. If the limit of detection was not met, the result was depicted as < 6.5 µg. These results (58%) where treated as 6.5/2 µg in the statistical calculations.

### 2.5. Analysis by XRD

A PanAnalytical diffractometer was used (PanAnalytical X’Pert Pro PW 3040/60, 2012). The system comprised of a generator, a vertical goniometer, a sample spinner, a graphite monochromator, an amplifier, a proportional counter, and a copper anode X-ray tube. Programmable divergence and receiving slits were used. Membrane filters were mounted in the diffractometer and drawn tight with a concentric ring holder. The samples were analyzed at a tube power of 45 kV and 40 mA. Diffraction line intensity was measured with an integrator, while the spectra was scanned at an angle rate of 0.033°/100 s. The α-quartz diffraction lines 4.26 Å, 3.34 Å and 1.82 Å appeared at 2θ angles of 20.85°, 26.67° and 50.15°, respectively. The integrator subtracted the background intensity using numerical peak fitting and interpolation. The net peak area reported by the instrument was alternatively used as the diffraction line intensity. A silicon blank was used as an external standard to correct for long-term instrumental drift. Results below the quantitative limit of determination (5 µg) were depicted as <5 µg. These results were treated as 5/2 µg in the statistical calculations.

### 2.6. Estimating the Amount of Workers Exposed to Respirable Crystalline Silica

Estimates on the number of workers exposed in different occupations presented in this study were based on the FINJEM job-exposure matrix [24]. The basic dimensions of exposure assessment in FINJEM are occupations, agents, and calendar periods. The number of workers employed by occupation and industry is based on data from the Central Statistical Office of Finland while the proportion of workers exposed within each occupation is-based in the expert judgement of 20 occupational hygiene professionals at FIOH. FINJEM includes 43 chemical exposures including quartz and 311 occupational categories further subdivided into 2–9 industrial subcategories [25].

### 2.7. Estimating the Amount of Workers with Silicosis or Cancers Attributable to Quartz Exposure

It is the duty of the employer to compensate workers for loss of earnings, health care costs, and disability due to an occupational accident or disease [26]. To bear this responsibility, employers are obliged to insure their employees against these. The Occupational Accidents Insurance Act has priority over other social insurance compensation systems. As part of this system, occupational diseases are diagnosed and verified at FIOH, based on clinical criteria and estimates on exposure during the working history of the employee. Workers that have a verified occupational disease are recorded in a register of occupational diseases in Finland. This register was used in the present study to estimate the number of silicosis cases per year.

### 2.8. Estimating Statistical Significance of the Trend in Respirable Quartz Exposure from 2013 to 2017

**Figure 1:**
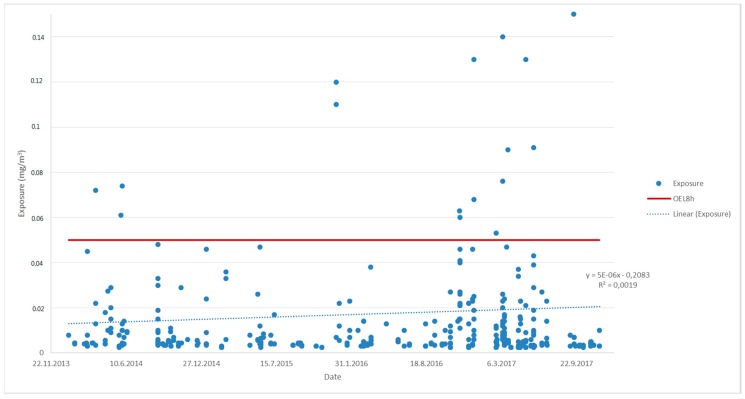
Exposure to respirable quartz in workplace measurements during the years 2014–2017.

A linear regression analysis was performed on the sampling date (x) and exposure (y) data (Figure 1). The Pearson correlation coefficient was tested against its sample-size dependent *p*-value (critical value, 95% level of significance, two tailed test) as described by Warner [27]: ρ_x,y_ = √(*t*^2^/(*t*^2^ + n − 2)) at the *t*-test critical value (*t*) corresponding to the relevant degree of freedom (*p* < 0.05). The test was performed to distinguish whether the exposure increased or decreased as a function of the date of sampling during the time-interval of the measurements.

## 3. Results

### 3.1. Measured Exposure (2006–2017)

**Figure 2:**
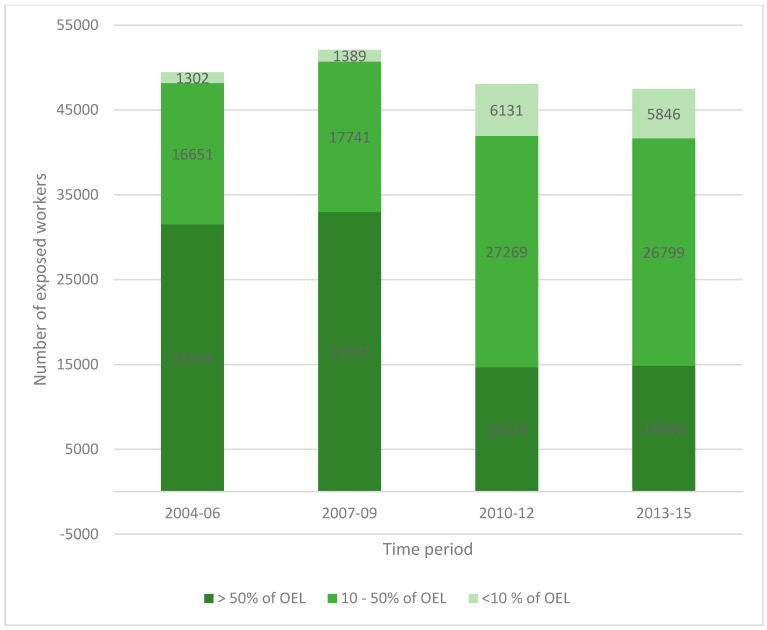
Number of workers estimated to be exposed to respirable quartz in Finland during 2004–2015.

For an eight-year period from the beginning of 2006 up until the end of 2013, the exposure to respirable silica in the light of the workplace measurements declined steadily [7]. Each year the average exposure was lower than during the previous year, as was the median exposure and the 95-percentile exposure except for years one and two, respectively (Table 1). However, from the beginning of 2013 to the end of 2017, the exposure seems to have stagnated at a relatively high level. During this period, exposure was on average 22–50% of the OEL_8h_ with a median of 8–12% of the OEL_8h_ (Table 1). In fact, there seems to be a slight increase in exposure starting 2013, but the increase is not statistically relevant (*p* < 0.05, Figure 1). The same stagnation in exposure can be seen from the number of workers potentially exposed at different concentrations (Figure 2). Between 2004 and 2009, an estimated 63–64% of workers were exposed to amounts of respirable quartz exceeding 50% of the OEL_8h_, whereas this proportion had dropped to 31% during 2010–2012 and remained approximately the same between 2013 and 2015 (Figure 2).

### 3.2. Exposure in Different Occupations (2013–2015) and the Number of Exposed Workers from 1995 to 2015

**Figure 3:**
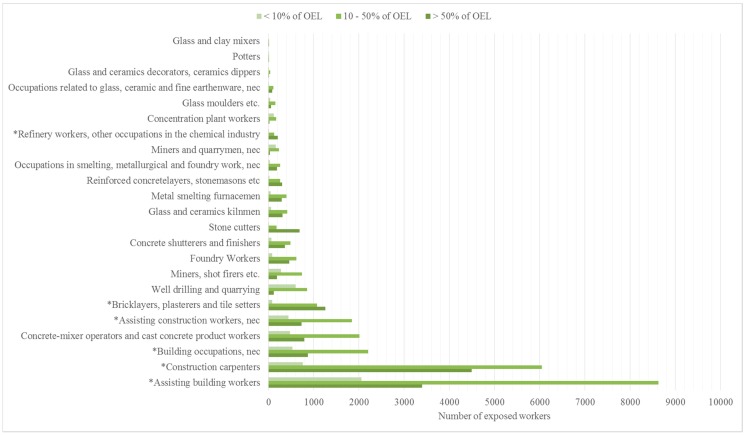
Respirable quartz concentrations in different occupations in relation to the OEL_8h_ during 2013–2015 according to FINJEM (* not formally covered by the NEPSI treaty).

Of the ten occupations where exposure to respirable quartz is presently highest, five is currently not covered by the NEPSI treaty. Particularly assistant building workers and construction carpenters are heavily exposed according to FINJEM (Figure 3). Occupations where the number of potentially exposed workers have been on the increase during recent years include construction work in general, particularly assisting building workers (Table 2). In addition, the number of exposed workers has increased in work tasks supporting building operations, such as in concrete cast production. Also, the opening of new mines in Finland has resulted in an increase in workers exposed to respirable silica in operations relating to mining and quarrying (Table 2). In most other occupations where respirable quartz exposure is of concern, the number of potentially exposed workers either declined or remained approximately the same from 2006 to 2015. One notable exception was refinery workers, among which an increase from 231 to 326 workers (45%) was recorded between 2006 and 2015 (Table 2).

### 3.3. Annual Incidence Rate and Annual Prevalence of Work-Related Silicosis and Lung Cancer from Respirable Quartz Exposure between 2005 and 2014

From 2005 to 2014, the number of verified occupational silicosis cases in Finland were on average 7.7 per year (Table 3), with a total of 85 cases. There is no visible upward or downward trend in the annual incidence rate during these years. The number of potentially exposed workers from 2004–2015 was on average 40,864 (Table 2), yielding an annual prevalence of silicosis of 2:10,000 workers (0.19‰). In other words, on average, 2 silicosis cases per 10,000 exposed workers were discovered each year. During the same time-period (2005–2014). From 2005 to 2014, lung cancers from respirable quartz exposure were diagnosed considerable less than silicoses. In all, five cancers were found, yielding a prevalence of 1:100,000 (0.011‰).

From 2005 to 2014, the incidence of occupational diseases related to respirable quartz exposure was highest in occupations related to mining (34%), building industries (28%), and foundry work and occupations in smelting and metallurgical work (15%) (Table 4). In addition, 9% of the silicosis or lung cancer cases were found in occupations supporting building industries (production of stone- and concrete products, cement, and tiles) and 8% in other occupations than those mentioned above (Table 4).

## 4. Discussion

The decline in respirable quartz exposure in Finnish workplaces between 2006 and 2013 after signing of the NEPSI treaty [7] was unique in extent. Few other agents during recent years, if any, come to mind where the work-related exposure has declined as steeply, while at the same time the number of workers exposed remained virtually the same. Clearly, the approach after the NEPSI treaty came into force in a good number of branches, concomitantly with a significant decrease of the OEL_8h_ from 0.2 to 0.05 mg/m^3^ was successful. In line with this, according to a recent survey, across the EU from 2007 to 2016 the level of exposure to respirable silica has decreased in most workplaces (73%), while the number of employees potentially exposed has decreased by over 50%, and more so in large companies with more than 250 employees [28].

Unfortunately, according to the present results, from 2013 onwards up until the end of 2017, the decline in Finland seems to have stagnated or have even been reversed. No further improvements were recorded in the present study, whether looking at the measured exposure, the number of potentially exposed workers in different branches, the branch-wise exposure in relation to the OEL_8h_, or the prevalence of silicosis and lung cancer from exposure to respirable quartz. The outbreak of silicosis or lung cancer in exposed workers in many cases takes more than ten years [17]. Consequently, it is conceivable that since quartz exposure declined significantly during the period from 2006 to 2013 in many branches, it is possible that in the future—within the next 10–20 years—we will see a decline in the number of silicosis and quartz-associated lung cancer patients in Finland. There is much room for improvement in this respect, particularly since all occupational diseases are not necessarily recorded and/or accepted by the insurance companies due to, for instance, lack of information about the exposure history or outside work factors contributing to the etiology of the established disease.

The respirable quartz exposure in many concerned branches seems to remain at a reasonably high level (on average 22–50% of OEL_8h_). Considering that an exposure of more than 50% of the current OEL_8h_ in Finland is associated with an increased risk of attaining silicosis or lung cancer [13,20,29]. In addition, since we have shown in the present study that the number of potentially exposed workers in branches not covered by the NEPSI treaty has increased during recent years, a decline in exposure as recorded during previous years (2006–2013) would require firstly an adjustment of the present OEL_8h_ in Finland to a level suggested by ACGIH and SCOEL, 0.02–0.025 mg/m^3^. Secondly, branches outside of the NEPSI treaty where the number of exposed workers is increasing in Finland, namely building and refinery industries, would be advised to sign the treaty.

## 5. Conclusions

As reported in an earlier study, application of good practices as described by the NEPSI agreement coincided with a strong decline in the exposure to respirable crystalline silica in Finnish workplaces during 2006 and 2013. In the present study, during the period 2013 to 2017 no further improvements were observed. The exposure remained at an average level of 20–50% of the current OEL_8h_, which is not necessarily sufficient to eliminate silicosis, lung cancer or other health effects associated with exposure to respirable silica in affected workplaces. Whether due to the latency period of the disease or the extent of exposure at workplaces, no improvements could be seen in the annual incidence rate or annual prevalence of silicosis in exposed workers during the years studied.

To bring about further improvements in exposure, we suggest the present OEL_8h_ in Finland (0.05 mg/m^3^) and particularly in the many EU countries with an OEL_8h_ of 0.1 mg/m^3^ be lowered to 0.020–0.025 mg/m^3^. Secondly, branches outside of the NEPSI treaty where the number of exposed workers is increasing in Finland and possibly in many other EU countries as well, namely building and refinery industries, would be advised to sign the treaty. In addition, as a result, good practices in these industries for work tasks where exposure to respirable silica is common should be developed.

## Figures and Tables

**Table 1:** Exposure to respirable quartz in workplace measurements during the years 2007–2017.

Year	Number of Samples	Average (mg/m^3^)	Median (mg/m^3^)	95% Percentile (mg/m^3^)	Nr. of Results > OEL_8h_
^1^ 2006	99	0.2036	0.0340	1.0100	44
^1^ 2007	71	0.0365	0.0100	0.1400	15
^1^ 2008	276	0.0443	0.0090	0.1325	41
^1^ 2009	155	0.0213	0.0065	0.0586	13
^1^ 2010	102	0.0158	0.0055	0.0596	8
^1^ 2011	197	0.0656	0.0055	0.1720	33
^1^ 2012	195	0.0180	0.0040	0.0522	11
^1^ 2013	44	0.0134	0.0058	0.0430	2
2014	87	0.0111	0.0050	0.0414	3
2015	67	0.0252	0.0040	0.0911	4
2016	95	0.0163	0.0060	0.0502	5
2017	158	0.0181	0.0050	0.0781	10

OEL_8h_ from 2007 onwards (0.05 mg/m^3^) was used throughout; ^1^ These breathing zone samples were included in the previously published results on workplace concentrations [7] but have not been published independently from measurements from fixed points.

**Table 2:** Workers exposed to respirable silica according to FINJEM-exposure matrix.

	The Number of Exposed Workers Per Time-Period						
Occupation	^2^ 1995–1997	^2^ 1998–2000	^2^ 2001–2003	^2^ 2004–2006	^2^ 2007–2009	2010–2012	2013–2015
^1^ Construction carpenters	7563	11,262	11,307	12,115	12,374	11,602	11,299
^1^ Assisting building workers	3802	5704	6745	8826	9568	15,732	14,071
^1^ Building occupations, nec	3469	3569	5214	6420	7439	2961	3597
^1^ Assisting construction workers, nec	1756	2633	3115	4075	4417	2798	3010
^1^ Bricklayers, plasterers, and tile setters	2495	3334	3347	3586	3662	2309	2415
Concrete-mixer operators and cast concrete product workers	1771	2207	2401	2379	2960	2774	3279
Foundry Workers	1496	2049	2125	2381	1888	1382	1153
Well drilling and quarrying	588	883	725	1617	1726	2061	1559
Metal smelting furnacemen	1813	1812	1810	1255	1427	634	736
Stone cutters	766	1080	1353	1012	1154	654	865
Miners, shot firers etc.	678	606	746	704	1089	953	1199
Occupations in smelting, metallurgical and foundry work, nec	898	923	905	1354	920	408	475
Glass molders etc.	679	953	943	1108	746	258	241
Reinforced concrete layers, stonemasons etc.	487	651	654	700	715	436	577
Concrete shutterers and finishers	524	587	669	507	647	899	899
Miners and quarrymen, nec	551	701	433	456	321	422	1199
Potters	218	245	201	301	268	258	17
Occupations related to glass, ceramic, and fine earthenware, nec	310	217	201	109	240	219	196
^1^ Refinery workers, other occupations in the chemical industry	257	280	189	241	231	308	336
Concentration plant workers	91	136	139	177	214	206	306
Glass and ceramics decorators, ceramics dippers	148	162	88	94	53	31	59
Glass and ceramics kilnmen	102	74	118	39	31	910	774
Glass and clay mixers	16	11	11	6	13	17	2
Sum of all occupations (total number of workers)	30,478	40,079	43,439	49,462	52,103	48,232	48,264
Sum of workers covered by the NEPSI treaty	11,136	13,297	13,522	14,199	14,412	12,522	13,526

^1^ Not formally covered by the NEPSI treaty; ^2^ These numbers have been previously published [7].

**Table 3:** The number of occupational diseases from respirable quartz exposure per year 2005–2014.

Year	Silicosis	Lung Cancers
2005	8	0
2006	14	0
2007	9	1
2008	10	1
2009	5	0
2010	5	0
2011	8	2
2012	8	1
2013	5	0
2014	11	0
Sum	84	5
Average	7.7	0.45
Annual prevalence	2:10,000	1:100,000

**Table 4:** The incidence of occupational diseases from different occupations between—and including—2005 and 2014.

Occupations	Incidence
Miners; shot firers and quarrymen in mining	30
Shot firers in building industries	6
Painters; carpenters; assistant building workers; plumbers; percussion drillers and deep drillers; assisting building and construction workers; other occupations in building industries	19
Foundry workers; occupations in smelting and metallurgical work	13
Welders, boilermakers, metal workers	6
Production of stone- and concrete products, cement, and tiles	8
Others	7
Sum	89

## References

[1] Klein C. (1993). Rocks, Minerals, and a Dusty World. Rev. Mineral..

[2] The European Network on Silica (NEPSI) (2013). Agreement on Workers Health Protection through the Good Handling and Use of Crystalline Silica and Products Containing it. Annex 1, Good Practices (Good Practice Guide).

[3] International Agency for Research on Cancer (IARC) (2012). IARC Monographs on the Evaluation of the Carcinogenic Risk of Chemicals to Humans. A Review of Human Carcinogens. Arsenic, Metals, Fibres, and Dusts.

[4] Official Statistics of Finland (OSF) (2018). Labor Force Survey.

[5] The Ministry of Economic Affairs and Employment in Finland (MEAE) (2018). Business Sector Services, Sector Reports, Mining Sector.

[6] The European Network on Silica (NEPSI) (2013). Agreement on Workers Health Protection through the Good Handling and Use of Crystalline Silica and Products Containing It.

[7] Tuomi T., Linnainmaa M., Väänänen V., Reijula K. (2014). Application of good practices as described by the NEPSI agreement coincides with a strong decline in the exposure to respiratory crystalline silica in Finnish workplaces. Ann. Occup. Hyg..

[8] CEN (Comite Europe’n de Normalisation) (1993). Workplace Atmospheres: Size Fraction Definitions for Measurements of Airborne Particles.

[9] ISO (International Standards Organisation) (1995). Air Quality—Particle Size Fraction Definitions for Health-Related Sampling.

[10] International Agency for Research on Cancer (IARC) (1997). Monographs on the Evaluation of the Carcinogenic Risk of Chemicals to Humans. Silica, Some Silicates, Coal Dust and Para-Aramid Fibres.

[11] National Institute of Occupational Safety and Health (NIOSH) (2002). Hazard Review: Health Effects of Occupational Exposure to Respirable Crystalline Silica.

[12] Saffiotti U. (2005). Silicosis and Lung Cancer, a fifty-year perspective. Acta Biomed..

[13] SCOEL (2003). Recommendation from the Scientific Committee (SCOEL) on Occupational Exposure Limits for Silica, Crystalline (Respirable Dust).

[14] Desphande A., Narayanan P., Lehnert B. (2002). Silica-induced generation of extracellular factor(s) increases reactive oxygen species in human bronchial epithelial cells. Toxicol. Sci..

[15] Polimeni M., Gazzano E., Ghiazza M., Fenoglio I., Bosia A., Fubini B., Ghizo D. (2008). Quartz Inhibits Glucose 6-Phosphate Dehydrogenase in Murine Alveolar Macrophages. Chem. Res. Toxicol..

[16] Knaapen A., Borm P., Albrecht C., Schins R. (2004). Inhaled particles and lugn cancer. Part A: Mechanisms. Int. J. Cancer.

[17] European Commission (EC) (1994). Information Notices on Diagnosis of Occupational Diseases.

[18] Duffin R., Gilmour P., Schins R., Clouter A., Guy K., Brown D., MacNee W., Borm P., Donaldson K., Stone V. (2001). Aluminium lactate treatment of dq12 quartz inhibits its ability to cause inflammation, chemokine expression, and nuclear factor-kappab activation. Toxicol. Appl. Pharmacol..

[19] Fubini B., Fenoglio I., Elias Z., Poirot O. (2001). Variability of biological responses to silicas: Effect of origin, crystallinity, and state of surface on generation of reactive oxygen species and morphological transformation of mammalian cells. J. Environ. Pathol. Toxicol. Oncol..

[20] Occupational Health and Safety Administration (OSHA) (2016). Frequently Asked Questions: Respirable Silica Rule.

[21] Kalberlah F., Bloser M., Wachholz C. (2005). Toleranz-und Akzeptanzschwelle für Gesundheitsrisiken am Arbeitsplatz.

[22] European Chemicals Agency (ECHA) (2012). Guidance on Information Requirements and Chemical Safety Assessment Chapter 8: Characterisation of Dose [Concentration] Response for Human Health.

[23] NIOSH (National Institute of Occupational Safety and Health) (2003). Silica, Crystalline by IR (KBr Pellet).

[24] Kauppinen T., Toikkanen J., Pukkala E. (1998). From Cross-tabulations to Multipurpose Exposure Information Systems: A New Job-Exposure Matrix. Am. J. Ind. Med..

[25] Pukkala E., Huo J., Kyyrönen P., Lindbohm M.-L., Sallmén M., Kauppinen T. (2005). National Job-Exposure Matrix in Analyses of Census-Based Estimates of Occupational Cancer Risk. Scand. J. Work Environ. Health..

[26] World Health Organization (WHO) (2012). Regional Office for Europe. National Profile of Occupational Health System in Finland.

[27] Warner R.M. (2013). Applied Statistics. From Bivariate to Multivariate Techniques.

[28] European Commission (EC) (2016). Study on the Implementation of the Autonomous Agreement of Workers’ Health Protection through the Good Handling and Use of Crystalline Silica and Products Containing It.

[29] Health and Safety Executive (HSE), Workplace Health Expert Committee (WHEC) (2017). Silica and Lung Cancer.

